# ApoCanD: Database of human apoptotic proteins in the context of cancer

**DOI:** 10.1038/srep20797

**Published:** 2016-02-10

**Authors:** Rahul Kumar, Gajendra P. S. Raghava

**Affiliations:** 1 Bioinformatics Centre, CSIR-Institute of Microbial Technology, Chandigarh-160036, India

## Abstract

In the past decade, apoptosis pathway has gained a serious consideration being a critical cellular process in determining the cancer progression. Inverse relationship between cancer progression and apoptosis rate has been well established in the literature. It causes apoptosis proteins under the investigative scanner for developing anticancer therapies, which certainly got a success in the case of few apoptosis proteins as drug targets. In the present study, we have developed a dedicated database of 82 apoptosis proteins called ApoCanD. This database comprises of crucial information of apoptosis proteins in the context of cancer. Genomic status of proteins in the form of mutation, copy number variation and expression in thousands of tumour samples and cancer cell lines are the major bricks of this database. In analysis, we have found that TP53 and MYD88 are the two most frequently mutated proteins in cancer. Availability of other information e.g. gene essentiality data, tertiary structure, sequence alignments, sequences profiles, post-translational modifications makes it even more useful for the researchers. A user-friendly web interface is provided to ameliorate the use of ApoCanD. We anticipate that, this database will facilitate the research community working in the field of apoptosis and cancer. The database can be accessed at: http://crdd.osdd.net/raghava/apocand.

Apoptosis is a crucial process in deciding the cell fate and received enormous attention among the biologists in the past decade^1^,^2^. Dysregulation of apoptosis network can lead to the pathological conditions and cancer is one of them^3^,^4^. Many studies have been done in the past to show the relation between apoptosis and cancer progression^5^,^6^,^7^. Not only cancer establishment and progression, apoptosis dysregulation also imparts drug resistance to almost all types of cancer^8^,^9^,^10^. Cancer molds expression levels of crucial apoptosis proteins in such a way that apoptosis suppressed, which helps in prolongation of cancer survival^11^,^12^. Expression level of anti-apoptotic proteins e.g. IAP proteins^13^,^14^,^15^ is increased and expression level of apoptotic initiator is decreased in cancer e.g. Caspases^16^,^17^,^18^ and SMAC/DIABLO^19^,^20^,^21^. Because of this many apoptosis proteins are considered as target of anticancer therapies to ameliorate the cancer treatment^22^,^23^,^24^,^25^. Moreover, few molecules targeting apoptosis proteins have entered into the clinical trials also^26^,^27^,^28^. To make anticancer therapy against apoptosis proteins more pragmatic, we need a database that comprises of substantial background information about these proteins in cancer e.g. genomic information, gene essentiality data, structure etc. A database of apoptosis proteins was developed by Diez *et al*. in 2010 called DeathBase^29^. This database covers many aspects of apoptosis proteins like sequence and structure information, evolutionary information, ontology and direct links to the other databases. On the other side, DeathBase lacks the genomic information e.g. mutations, copy number variation and expression, gene essentiality, which is a preliminary requirement to understand these proteins in the context of cancer. It also does not provide any information regarding tertiary structure of these proteins. So, DeathBase is implausible for studying the apoptosis proteins in the context of cancer. For the better understanding of apoptosis proteins in cancer, still there is great demand for dedicated database of apoptosis proteins, which compiles information regarding their genomic status in large number of tumour samples and cancer cell lines along with the tertiary structure and gene essentiality data at one platform. With this tenet in mind, we have developed a dedicated database called ApoCanD, which comprises of compelling information about mutations, copy number variation, expression, gene essentiality, modeled tertiary structure, sequence alignment, sequences profiles from position-specific scoring matrix (PSSM) and hidden Markov models (HMM), structure domains, post-translational modification (PTM) and direct links to essential databases. Information in ApoCanD will complement the research community in designing anticancer therapies against apoptosis proteins.

## Database Aim

ApoCanD is dedicated to understanding the role of apoptosis proteins in the cancer progression and drug resistance development. To address this issue, we have compiled three types of genomic information of apoptosis proteins e.g. mutation status, copy number variation and gene expression levels in tumour samples and cell lines. Along with this, normal variations from 1000 genome project^30^ are also compiled, which allow comparative benchmarking of normal and cancer samples. Gene expression and copy number variation data of these proteins is also included to understand their role in cancer. All the proteins are not essential for the survival of a particular cancer, so we have included the gene essentiality data in ApoCanD. Other basic information like, their tertiary structure, sequence alignment and profiles, post-translation modification etc. on a user-friendly web platform to draw fundamental conclusions about cancer. This web platform will provide plenty of opportunities for the researchers in exploring the role of apoptosis in cancer.

## Backend Information

ApoCanD is built on Apache HTTP server, which is platform independent and available as open-source software. MySQL database is used to store the information in backend. Front end is developed by PHP, HTML, CSS and Java integration. Perl is also used as programming language to process the data in web presenting form.

## Content of Database

**Figure 1:**
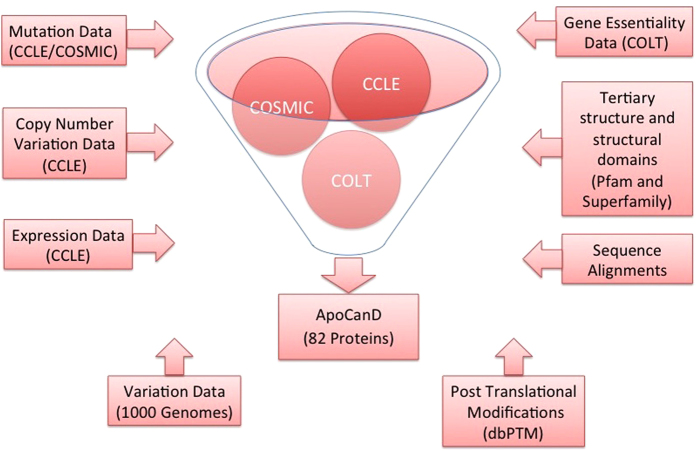
Schematic diagram showing the curation procedure of ApoCanD.

ApoCanD contains various types of information about each apoptosis protein, which is collected from different resources. Figure 1 shows the procedure of curation of ApoCanD.

### Apoptosis Genes

**Figure 2:**
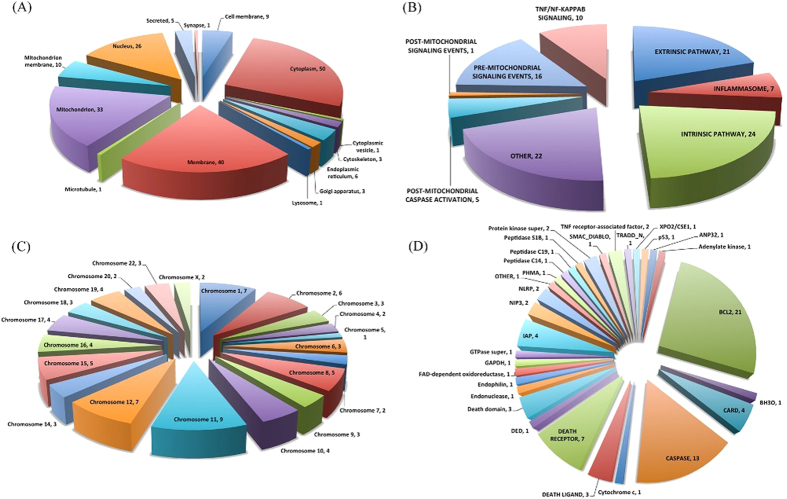
Distribution of apoptosis proteins by various categories (**A**) Cellular location (**B**) Pathway (**C**) Chromosomal distribution and (**D**) Protein family.

We have followed the same criteria adopted in the DeathBase^29^ for the selection of apoptosis proteins e.g. they should be the part of the core machinery of apoptosis, they should regulate the core machinery of apoptosis, proteins having characteristic apoptotic domains or they are homologous to the central proteins of apoptosis. Following these criteria, we have selected 82 proteins and done our study on these proteins for the development of ApoCanD. Figure 2 represents the distribution of these proteins by various categories e.g. cellular location, pathway, chromosomal location and protein family.

### Mutation Data

**Figure 3:**
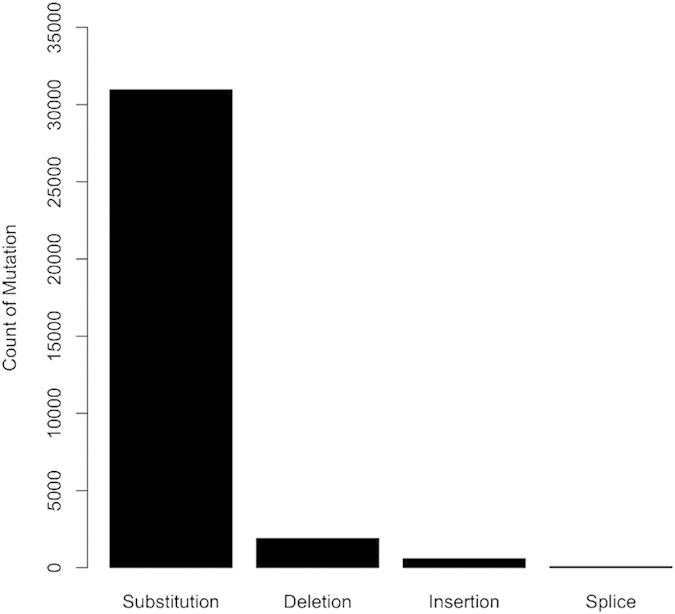
Bar graph showing the count of mutation types.

Mutation data of each apoptosis protein is collected from two primary resources namely, Cancer Cell Line Encyclopedia (CCLE), released on 24-Oct-2012 (*CCLE_hybrid_capture1650_hg19_NoCommonSNPs_NoNeutralVariants_CDS_2012.05.07.maf*)^31^ and Catalogue of Somatic Mutation in Cancer (COSMIC), version 67 (*CosmicMutantExport_v67_241013.tsv*) for tumour samples and cell line mutation data *(CosmicCLP_MutantExport.tsv)*^32^. CCLE contains the mutation status of more than 1600 proteins in 947 cancer cell lines, from this data we have filtered out the total 1368 mutations of apoptosis proteins. Similarly, we have filtered out 32157 mutations of apoptosis proteins available in cosmic data, which contains mutation data from both tumour samples and cancer cell lines. Out of the total mutations of 33525, major type of mutation is substitution mutation (30957) as shown in Figure 3.

### Gene Expression Data and Copy Number Variation

Copy number variation and gene expression data of apoptosis proteins were collected from the CCLE. In CCLE, expression data was obtained from Affymetrix U133 plus array and further normalized by RMA technique using quantile normalization. Similarly, copy number variation data was obtained from Affymetrix SNP 6.0 arrays.

### Gene Essentiality Data

**Figure 4:**
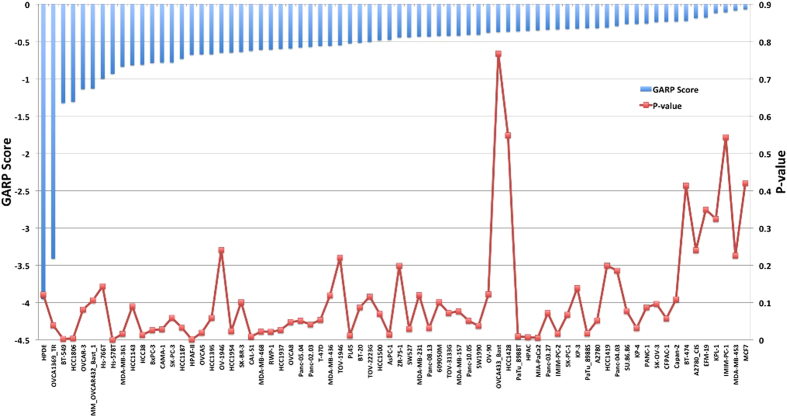
Gene essentiality data of X-linked inhibitor of apoptosis protein (XIAP) in different cancer cell lines.

Every gene is not required for the survival, so it is essential to check the importance of each gene in survival of cancer cells. So, we have compiled the shRNA dropout profiles of apoptosis genes from COLT-cancer database^33^. In COLT-cancer database, gene essential profiles of approximately 16000 genes are present, which is identified in 72 breast, ovarian and pancreatic cancer cell lines. Gene essentiality data is given in the form of two parameters. First GARP score, which quantifies the shRNA dropout rate, lower GARP score (more negative) represents the high essentiality of that gene. Second parameter is P-value, which describes the significance of GARP score. Figure 4 represents the gene essentiality data of XIAP in 72 cancer cell lines, where it shows the essentiality of this gene for HPDE and OVCA1369 cell lines.

### Tertiary Structure

Structural information is essential for the designing of targeted therapy against any protein target. So, we found out the crystal structure of apoptosis proteins in PDB and out of 82 apoptosis proteins, structures of 61 proteins were available in PDB. Although for most of the proteins, structures were available in PDB, still we modeled the structure of all the proteins using HH-suite 2.0.16^34^ and Modeller 9.13^35^. Modeled structure of each protein can be visualized in Jmol applet and their respective PDB files can be downloaded by clicking on download button. We have also provided the PDB IDs of known structures and directly linked them to the PDB website.

### Structure Domains

Many of the apoptosis proteins contain characteristic domains e.g. BIR domains in IAPs proteins. We have mapped the Pfam^36^ and Superfamily^37^ domains in all the apoptosis proteins. PFAM domains were searched by querying at Pfam website using the fasta sequences and E-value were kept at default value of 1. For Superfamily domains, we used standalone version of Superfamily and ran it with default parameters. We got 233 Pfam domains and 139 Superfamily domains in 82 apoptosis proteins.

### Sequence Alignment

In ApoCanD, we have generated four types of sequence alignments of apoptosis proteins. First, alignment with variants obtained from 1000 genome project. In 1000 genome project, VCF file of more than 1000 genomes are given. We have converted the VCF file format to ANNOVAR input and then extracted the respective variations for each protein using ANNOVAR software^38^. These variations were mapped on to the wild-type protein sequences and these variants were aligned with wild-type sequences. Second, alignment with CCLE mutants, here mutants available in cancer cell lines were obtained from the CCLE and aligned with wild-type sequences. Third, alignments were generated with cancer mutants available in COSMIC. Fourth, we have obtained the homologous proteins of each human apoptosis proteins in other species from the NCBI and aligned them with human apoptosis proteins. In the case of homologous proteins, we have also generated the evolutionary tree. For the sequence alignment, we have used ClustalW^39^ and for the better visualization of sequence alignments and evolutionary tree, Jalview^40^ was used.

### Sequence Profiles

Two types of sequence profiles were generated and included in ApoCanD e.g. HMM and PSSM, which tells about the conservation score at each position of the protein. HMM profiles were made by using ‘jackhmmer’ and ‘hmmbuild’ modules of HMMER software^41^ and PSSM profiles were generated by the ‘blastpgp’ and ‘makemat’ module of BLAST software^42^. We have created these alignments with three types of sequence databases e.g. Uniprot database, mutated sequences database (CCLE and COSMIC) and normal variant database (1000 Genomes). HMM profiles with mutants were proved to be excellent in predicting the functional impact of a mutation^43^, so we generated them for each apoptosis protein.

### Post-Translational Modifications

Post-translational modifications (PTMs) play a crucial role in the functioning of proteins^44^,^45^,^46^. So, we included the PTM information in ApoCanD, which were compiled from the dbPTM^47^. In ApoCanD, position of the modification, amino acid (where modification occurred) and type of modification is given for each apoptosis protein. Major types of PTMs are phosphorylation, acetylation and ubiquitylation.

## Querying the Database

For the maximal use of ApoCanD data with ease, we have provided three methods to query or access the database.

### Tools

In tools section, we have provided five modules to access the data. *(i) Simple search:* Here user can query the data by entering a simple keyword e.g. protein name, cellular location, pathway, domain name, family etc. Simple search returns an aesthetic table containing all the major information available about the query keyword. *(ii) Advanced search:* It allows searching on the basis of three fields e.g. cellular location, pathway and family. Advance search uses logical operators (AND/OR) in returning the final result. ‘All’ option is given in all the three fields, which represents the OR function. Selecting ‘All’ option in all the three fields returns all the information about 82 proteins available in ApoCanD. *(iii) BLAST:* Here user can do the BLAST of a query protein with the apoptosis proteins available in ApoCanD to see the similarity of query protein with apoptosis proteins. *(iv) Alignment:* Here user can do the alignment of their query sequence with four different types of protein sequences, *(a)* With wild type apoptosis protein sequences, *(b)* With CCLE mutants, *(c)* With COSMIC mutants and *(d)* With 1000 Genome variants. Alignment can be visualized on Jalview applet, which gives three types of information *i.e.* conservation, quality and consensus along with the protein sequence alignment. This module also provides the option to view the dendrogram tree of query sequence with the respective protein type selected. *(v) Cancer Sensitivity:* This tool helps users to predict the nature of a protein sequence change, whether it would be a normal variation or a cancer sensitive mutation. This tool is based on the similarity score of the query sequence with the HMM profile of normal variants from 1000 Genome project and the cancer mutants from CCLE and COSMIC. Altered query sequence will be declared as cancer sensitive, if the similarity score is higher with cancer mutants HMM profile and vice versa. This tool uses “hmmsearch” module of HMMER suite (version 3.1b1)^48^ for calculating the similarity with respective HMM profiles.

### Browse

Browse section has four inbuilt modules, which will help users to browse the ApoCanD database to draw the substantial information regarding apoptosis pathway proteins. *(i) Protein:* This module enlists all the 82 apoptosis proteins in a single aesthetic table and each protein is linked to its respective summary page. This table consists of five other fields apart from the gene name, Uniprot link, Deathbase link, homologues proteins link, PDB link and PubChem/ChEMBL bioassay link. Clicking on any of them opens up the respective information page on the host website. *(ii) Chromosomes:* In this module, apoptosis proteins have been divided on the basis of their gene location on their respective chromosomes. Here, user can browse the apoptosis proteins on the basis of the chromosomes distribution of their genes. Chromosome distribution of apoptosis genes is also depicted by the Circos plot, which also shows the gene expression and copy number variations (CNV) of apoptosis genes. *(iii) Mutation Source:* Mutational information of apoptosis genes was taken from CCLE and COSMIC and normal variants were taken from 1000 Genome project. Then, we identified the frequencies of each protein for cancer mutation and normal variation. Further, we calculated the ratio of the cancer mutation frequency and normal variant frequency for each protein, which built the list of highly mutated proteins in cancer and could be a target for anticancer therapy. TP53 has the highest frequency of mutation in cancer, which is approximately 1100 times more mutated in cancer as compared to the normal (Table 1). Second most mutated protein (100 times) is MYD88, which is an adapter protein involved in Toll-like receptor signaling pathway^49^. This protein is a well-established target for cancer therapy, whose mutation is responsible for the proliferation of cancer cells^50^. This module will help users to identify such apoptosis proteins, which are more prevalent for mutations in cancer and could be a target for anticancer therapy*. (iv) Genomic Features:* Here, we allow the users to select the apoptosis proteins on the basis of their genomic features *i.e.* mutation frequency, gene expression and copy number variation (CNV). User can select a particular range available in the option to fish out the apoptosis proteins.

### Pathway

**Figure 5:**
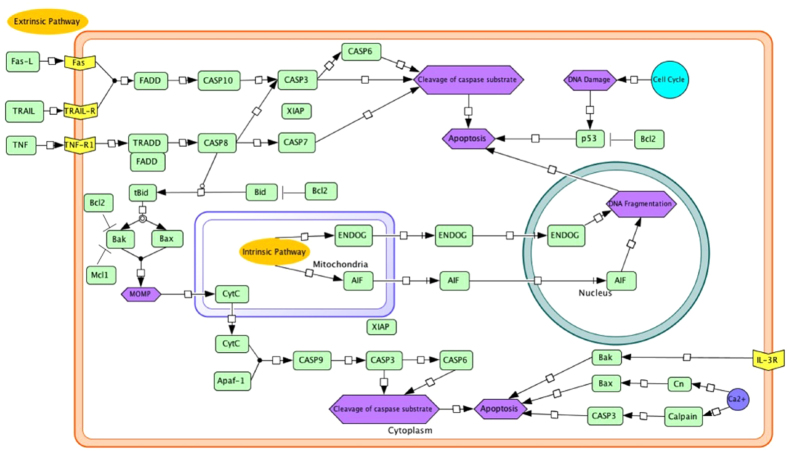
Diagrammatic view of apoptosis pathway.

On this page, diagram of apoptosis pathway (intrinsic and extrinsic) is provided, which is designed by the CellDesigner software^51^ and adopted from the KEGG pathways^52^ as shown in Figure 5. To keep it simple, we have linked this pathway diagram with respective summary page of each protein. User can just click on a protein to open its summary page. At the end of the page, legends of pathway diagram are given to understand the pathway diagram.

## Information

This section is dedicated to the relevant information about ApoCanD, which provides the following four type of information, *(i) Statistics*: This page summarizes the statistics about the distribution of ApoCanD proteins i.e. family wise, pathway wise, cellular location wise, frequency of mutation types and chromosome wise. *(ii) Publication*: This page provides the list of most recent papers about the apoptosis available in PubMed. *(iii) Related Links*: Here, user can find the relevant links of databases related to apoptosis. *(iv) Acknowledgment*: This page acknowledges the authors of databases and software used in the construction of ApoCanD.

## Downloads

To complement and growth of this research field, we have provided all the data of ApoCanD for download. A dedicated download page is built, which gives access to the user to download the data without any condition. Data available for download includes mutation, copy number variation, expression data; modeled tertiary structures and domains; sequence alignment and profiles (PSSM and HMM); post-translational modifications for all the apoptosis proteins available in ApoCanD.

## Discussion and Future Direction

**Figure 6:**
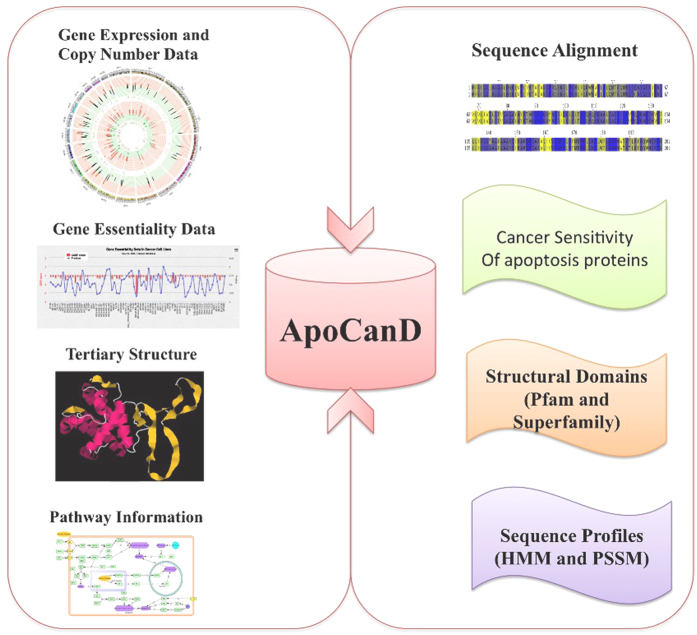
Various applications of ApoCanD database.

Apoptosis pathway has a crucial role in cancer survival and development of drug resistance against a number of anticancer drugs. It is an urgent need of the present time to understand the role and mechanism of apoptosis proteins in cancer. Database of apoptosis proteins developed earlier just compiled the information about these proteins, which is not enough to understand their role in cancer. So, we developed a dedicated database of apoptosis proteins in the present study, which is in the context of cancer. ApoCanD is a critical step in this direction, which compiles fundamental information required for apoptosis proteins to gain deeper insight into their role in cancer. Genomics data available in ApoCanD helps researchers to identify the mutation, copy number variation and expression level status of apoptosis proteins in thousand of tumour samples and cancer cell lines. For example, TP53 protein is mutated in most of the cancer cell lines; X-linked inhibitor of apoptosis proteins (XIAP) is over-expressed in most of the cancer cell lines and inhibits caspases, which helps cancer cells to inhibit apoptosis and grow without any regulatory check. On the other hand SMAC/DIABLO is under-expressed, which further suppresses apoptosis. There are many such relevant information can be drawn from the ApoCanD data. Further, gene essentiality data makes it convenient to find out, which gene is essential for the survival of a particular cancer cell line and could act as therapeutic target for drug development. Structure and sequence alignment information in ApoCanD makes it easier to look for a possible strategy to target a protein for the therapeutic point of view. Figure 6 illustrates the applications of ApoCanD. Mutation and expression data available in ApoCanD can be used to develop predictive features of drug response by applying machine learning algorithm like naïve bayes, elastic net, support vector machine etc^31^,^53^,^54^,^55^. Moreover, mutation and copy number variation data can be explored to predict cancer subtype-specific drug response^55^,^56^,^57^,^58^. Apoptosis is a critical pathway in cancer and found to be deregulated in cancer^5^,^6^,^7^ and explored for fishing cancer therapeutic targets^10^,^11^,^23^,^59^.Genomic data available in ApoCanD can be explored for designing map of apoptosis pathway, which can be used for assigning cancer associated apoptosis genes as biomarkers^60^. Hudson *et al*.^61^ shown a comparative study of mutation data from CCLE and COSMIC, where they found discrepancies in these two large mutation datasets. These discrepancies were attributed to differences in computational protocols e.g. dbSNP filtering, acquisition/loss of mutation, passaging of cell lines etc. Unfortunately, these factors cannot be avoided in such large genomic studies and sometime limit down the impact of such studies. But from somewhere at some point, we have to start our efforts to address the critical disease problems. In future, we will try to develop quantitative structure-activity relationship (QSAR) models for most of the apoptosis proteins to develop nifty chemical molecules against them. Integration of such models on ApoCanD platform will make it more useful for the researchers, where they can choose their drug target and design active molecules against them at one platform. We keep on increasing the quantity and quality of the data in ApoCanD to makes it a cutting edge tool for the researchers in the apoptosis field.

## Additional Information

**How to cite this article**: Kumar, R. and Raghava, G. P. S. ApoCanD: Database of human apoptotic proteins in the context of cancer. *Sci. Rep.* **6**, 20797; doi: 10.1038/srep20797 (2016).

## Figures and Tables

**Table 1:** Most mutated apoptosis proteins in cancer (Top 10).

S. No.	Protein	Uniprot Accession	C1	C2	C1 + C2	C3	C1 + C2/C3
1	TP53	P04637	627	19069	19696	17	1158.59
2	MYD88	Q99836	20	782	802	8	100.25
3	TNF	P01375	41	15	56	5	11.20
4	CASP8	Q14790	40	105	145	15	9.67
5	NLRP3	Q96P20	97	282	379	44	8.61
6	BMF	Q96LC9	9	18	27	4	6.75
7	BCL2L1	Q07817	16	22	38	6	6.33
8	FASLG	P48023	18	44	62	10	6.20
9	CASP3	P42574	8	27	35	6	5.83
10	BCL2L11	O43521	11	29	40	7	5.71

*C1: Mutation count in CCLE; C2: Mutation count in COSMIC; C3: Variant count in 1000 Genome variation.*
